# Eye-Blink Behaviors in 71 Species of Primates

**DOI:** 10.1371/journal.pone.0066018

**Published:** 2013-05-31

**Authors:** Hideoki Tada, Yasuko Omori, Kumi Hirokawa, Hideki Ohira, Masaki Tomonaga

**Affiliations:** 1 Department of Psychology, Tohoku-Gakuin University, Sendai, Japan; 2 Jin’ai University, Echizen, Japan; 3 Okayama University, Okayama, Japan; 4 Nagoya University, Nagoya, Japan; 5 Primate Research Institute of Kyoto University, Inuyama, Japan; Institut Pluridisciplinaire Hubert Curien, France

## Abstract

The present study was performed to investigate the associations between eye-blink behaviors and various other factors in primates. We video-recorded 141 individuals across 71 primate species and analyzed the blink rate, blink duration, and “isolated” blink ratio (i.e., blinks without eye or head movement) in relation to activity rhythms, habitat types, group size, and body size factors. The results showed close relationships between three types of eye-blink measures and body size factors. All of these measures increased as a function of body weight. In addition, diurnal primates showed more blinks than nocturnal species even after controlling for body size factors. The most important findings were the relationships between eye-blink behaviors and social factors, e.g., group size. Among diurnal primates, only the blink rate was significantly correlated even after controlling for body size factors. The blink rate increased as the group size increased. Enlargement of the neocortex is strongly correlated with group size in primate species and considered strong evidence for the social brain hypothesis. Our results suggest that spontaneous eye-blinks have acquired a role in social communication, similar to grooming, to adapt to complex social living during primate evolution.

## Introduction

Support for the social brain hypothesis has come from the fact that the neocortex is strongly correlated with group size in primate species [1]–[3]. Eye-blinks in humans are influenced by a variety of factors, including peripheral, central, and environmental factors [4]–[7]. In addition, many studies have identified marked interindividual differences in eye-blink behavior [8]–[10], although few have directly examined the origin of these differences. Most of these studies compared a pathological group with a healthy control group and showed that compared with the control groups, the blink rate is higher in individuals with schizophrenia or Huntington's chorea [11]–[15] and lower in individuals with parkinsonism or progressive supranuclear palsy [4], [7], [16]–[20].

Numerous developmental studies on human blinking have been performed [4], [16], [21]–[25]. Many of these studies confirmed the conclusions of Ponder and Kennedy [4] that blinking is virtually absent at birth. However, no consistent findings have been reported regarding the subsequent development of blinks. One study indicated that the blinking rate increased during development until adulthood [21], whereas another study showed that the peak rate of blinking occurred in preadolescence [24]. Nevertheless, both studies showed that after the peak, no significant differences in blink rates occur at older ages. However, another study in subjects from 5 to 87 years old indicated no differences in the blink rate with age [22].

The most systematic study of eye-blinking in nonhuman animals is that performed in 1927 by Blount [26], who observed blinking in 39 species, including amphibians and primates, and described their blinking behaviors in detail. His observations revealed marked changes in blinking behaviors. For example, the interblink interval was prolonged in dairy cows during feeding and neck licking, in pre-ruminating bison, and in wolves watching birds. Based on these results, Blount concluded that these blinks have a central rather than peripheral origin [26]. These results were consistent with those reported by Ponder and Kennedy [4], who focused on human blinking and suggested the importance of central processes. Blount also suggested that blink rates show an evolutionary history, albeit with some exceptions [26]. Using these results, we calculated the mean blink rate and standard deviation (SD) of 39 species [approximately 6.8 ± 9.1 blinks per minute (bpm)]. The coefficient of variation (CV  =  SD/mean × 100) was 134%, which suggested marked interspecies variations in blink rates.

Surprisingly, very few studies have determined the blink rates in animals. One series of studies investigated the dopamine hypothesis of blinking in animals [27]–[33]. Another series directly measured the blink rates in animals, including cows [34], birds [35], dogs [36], guinea pigs [37], rabbits [37], [38], and 31 mammalian species [13]. These studies confirmed that nocturnal animals blink much less frequently than diurnally active animals, blinking frequency being one-tenth lower in nocturnal versus diurnal mammals [13] and birds [35]. Carrington et al. [36] reported that the mean ± SD blink rate in dogs is 14.5 ± 5.63 bpm, and approximately 66% of all blinks were incomplete. Note that interindividual differences are quite large in dogs [36], but much smaller in birds [35]. Mowrer reported a detailed description of the nictitating membrane (i.e., the third eyelid) of birds [39].

Studies on blinking in nonhuman primates will help us to understand human eye behaviors. Several studies have focused on head-cocking behaviors in nonhuman primates [40] and the properties of the sclera in primates [41]. Several studies on animal blinking support this phylogenetic view [26], [34], [35], but no systematic investigations have examined nonhuman primates, the group of species most closely related to humans. Therefore, studies on nonhuman primates may be particularly important because previously reported studies did not distinguish among various types of blinks, including pseudo-blinks (nictitating membrane activities), half blinks, winks (one eye only, involuntarily blinks), and flickers [29], [39] and did not examine the relationship between eye-blink behaviors and other factors. Therefore, the present study was performed to examine the factors affecting blinking behaviors in other primates, the group most closely related to humans, with the most similar eye-blink behaviors.

## Methods

### Subjects

We video-recorded 141 individuals across 71 species of primates housed at the Japan Monkey Centre (JMC), Inuyama, Aichi, Japan, in addition to two gorillas (one male and one female) and three chimpanzees (one male and two females) housed at Yagiyama Zoological Park, Sendai, and two orangutans (one male and one female) housed at Chiba Zoological Park, Chiba, Japan. All of the species are listed in Table 1. Phylogenetic classification was determined on the basis of Rowe [42] and Groves [43].

**Table 1:** Data on the three measures of eye-blinking behavior, two body-size measures, and group size for each primate species.

No	Species	Scientific name	N	Family1)	Activity rhythms2)	Habitat types3)	Body weight in kg	Blink Rate in bpm	Blink Duration in ms	Isolated Blink Ratio in%	Group Size4)
1	Ring-tailed lemur	*Lemur catta*	1	Lem	D	A	2.8	0.4	231.0	0.0	13.8
2	Black-and-white ruffed lemur	*Varecia variegata*	1	Lem	D	A	3.8	3.7	323.4	15.4	8.0
3	Brown lemur	*Eulemur fulvus*	1	Lem	D	A	2.5	19.0	250.8	31.0	9.4
4	Black lemur	*Eulemur macaco*	2	Lem	D	A	2.4	2.0	194.7	18.8	10.0
5	Senegal bushbaby	*Galago senegalensis*	1	Lor	N	A	0.2	0.3	---	33.3	1.0
6	Brown greater galago	*Otolemur crassicaudatus*	2	Lor	N	A	1.3	0.3	341.0	0.0	1.0
7	Potto	*Perodicticus potto*	1	Lor	N	A	0.6	0.0	---	---	1.0
8	Lesser slow loris	*Nycticebus pygmaeus*	1	Lor	N	sA	0.8	0.2	---	100.0	1.0
9	Goeldi's marmoset	*Callimico goeldii*	2	Cal	D	A	0.5	9.1	141.9	17.9	8.0
10	White-headed marmoset	*Callithrix geoffroyi*	2	Cal	D	A	0.3	8.1	188.1	13.3	5.0
11	Common marmoset	*Callithrix jacchus*	2	Cal	D	A	0.3	5.4	178.2	12.6	9.5
12	Black-tufted marmoset	*Callithrix penicillata*	1	Cal	D	A	0.2	6.5	194.7	5.0	8.0
13	Pygmy marmoset	*Cebuella pygmaea*	2	Cal	D	A	0.1	4.3	212.9	23.2	5.5
14	Emperor tamarin	*Saguinus imperator*	2	Cal	D	A	0.4	8.1	209.6	17.7	4.0
15	White-lipped tamarin	*Saguinus labiatus*	2	Cal	D	A	0.4	10.2	198.0	19.2	7.5
16	Red-handed tamarin	*Saguinus midas*	2	Cal	D	A	0.5	14.2	203.8	8.5	5.0
17	Cotton-top tamarin	*Saguinus oedipus*	1	Cal	D	A	0.4	14.1	---	65.8	6.0
18	White-fronted spider monkey	*Ateles belzebuth*	3	Ceb	D	A	7.7	7.2	168.3	24.0	24.8
19	Geoffroy's spider monkey	*Ateles geoffroyi*	1	Ceb	D	A	7.7	16.3	287.1	24.3	31.0
20	Red-faced spider monkey	*Ateles paniscus*	3	Ceb	D	A	8.8	7.3	288.8	21.2	18.0
21	White-fronted capuchin	*Cebus albifrons*	2	Ceb	D	A	2.1	29.8	205.6	14.5	19.8
22	Tufted capuchin	*Cebus apella*	3	Ceb	D	A	3.1	5.9	181.5	34.3	18.0
23	White-headed capuchin	*Cebus capucinus*	4	Ceb	D	A	2.8	8.5	315.0	48.8	16.4
24	Brown wooly monkey	*Lagothrix lagotricha*	2	Ceb	D	A	5.9	9.9	298.7	38.5	31.8
25	Pale-headed (white-faced) saki	*Pithecia pithecia*	3	Ceb	D	A	1.8	5.4	173.3	16.2	4.4
26	Black-capped squirrel monkey	*Saimiri boliviensis*	1	Ceb	D	A	0.8	3.0	148.5	0.0	60.0
27	Dusky titi	*Callicebus moloch*	6	Ceb	D	sA	0.8	2.2	221.7	17.0	3.5
28	Owl monkey	*Aotus trivirgatus*	2	Ceb	N	A	0.9	3.4	290.4	13.5	2.9
29	Allen's swamp monkey	*Allenopithecus nigroviridis*	2	Cer	D	A	4.8	14.0	161.7	20.5	40.0
30	Agile mangabey	*Cercocebus agilis*	2	Cer	D	A	7.6	18.0	158.4	32.5	35.8
31	Red-tailed monkey	*Cercopithecus ascanius*	1	Cer	D	A	3.8	13.1	306.9	12.9	29.2
32	Schmidt's guenon	*Cercopithecus ascanius schmidti*	1	Cer	D	A	4.6	16.6	257.4	5.6	29.2
33	Moustached guenon	*Cercopithecus cephus*	1	Cer	D	A	3.5	22.0	161.7	12.9	6.0
34	Blue monkey	*Cercopithecus mitis albogularis*	2	Cer	D	A	5.8	8.3	148.5	26.0	26.8
35	Mona monkey	*Cercopithecus mona*	1	Cer	D	A	4.3	16.3	161.7	6.3	9.8
36	Mantled guereza	*Colobus guereza*	6	Cer	D	A	10.2	8.0	222.5	45.9	9.3
37	Black-and-white colobus	*Colobus polykomos angolensis*	2	Cer	D	A	8.6	7.2	206.3	38.4	13.6
38	Angolan Talapoin	*Miopithecus talapoin*	1	Cer	D	A	1.2	20.6	174.9	11.9	64.0
39	Proboscis monkey	*Nasalis larvatus*	1	Cer	D	A	15.3	8.8	231.0	39.4	12.7
40	Silvery lutung	*Trachipithecus cristatus*	2	Cer	D	A	7.0	17.3	287.1	37.4	35.0
41	Francois's langur	*Trachipithecus francoisi*	2	Cer	D	A	6.5	13.5	181.5	46.8	9.1
42	Golden-bellied mangabey	*Cercocebus chrysogaster*	2	Cer	D	sA	7.8	9.9	161.7	14.6	15.0
43	White-crowned mangabey	*Cercocebus torquatus lunulatus*	2	Cer	D	sA	5.2	7.9	191.4	43.2	37.0
44	De Brazza's guenon	*Cercopithecus neglectus*	1	Cer	D	sA	5.8	18.1	198.0	10.1	9.2
45	Vervet monkey	*Chlorocebus aethiops*	3	Cer	D	sA	3.9	10.0	178.2	15.9	40.5
46	Formosan macaque	*Macaca cyclopis*	1	Cer	D	sA	7.0	16.7	194.7	34.4	46.8
47	Long-tailed macaque	*Macaca fascicularis*	1	Cer	D	sA	5.3	10.3	155.1	22.2	20.0
48	Japanese macaque	*Macaca fuscata*	1	Cer	D	sA	12.3	15.1	234.3	61.3	40.3
49	Yaku macaque	*Macaca fuscata yakui*	1	Cer	D	sA	11.6	5.1	178.2	20.8	40.3
50	Rhesus macaque	*Macaca mulatta*	1	Cer	D	sA	7.0	10.5	198.0	18.7	56.2
51	Pig-tailed macaque	*Macaca nemestrina*	2	Cer	D	sA	7.4	17.2	163.4	28.9	44.5
52	Bonnet macaque	*Macaca radiata*	1	Cer	D	sA	5.2	20.0	171.6	23.4	19.1
53	Lion-tailed macaque	*Macaca silenus*	2	Cer	D	sA	6.2	9.9	181.5	4.9	32.3
54	Toque macaque	*Macaca sinica*	2	Cer	D	sA	4.8	12.6	189.8	22.0	24.8
55	Barbary macaque	*Macaca sylvanus*	4	Cer	D	sA	13.0	19.5	163.4	47.4	49.7
56	Tibetan macaque	*Macaca thibetana*	3	Cer	D	sA	13.2	22.6	290.4	68.9	38.3
57	Patas monkey	*Erythrocebus patas*	3	Cer	D	T	7.9	4.1	190.3	15.0	34.8
58	Drill	*Mandrillus leucophaeus*	2	Cer	D	T	13.5	11.1	222.8	31.6	96.5
59	Anubis (Olive) baboon	*Papio anubis*	1	Cer	D	T	21.6	18.0	---	46.4	50.0
60	Hamadryas baboon	*Papio hamadryas*	1	Cer	D	T	14.5	17.5	290.4	54.0	80.5
61	Guinea baboon	*Papio papio*	2	Cer	D	T	8.8	11.1	138.6	49.3	40.5
62	Hanuman langur	*Presbytis entellus*	1	Cer	D	T	15.4	18.2	214.5	34.4	45.0
63	Gelada baboon	*Theropithecus gelada*	3	Cer	D	T	13.8	11.9	248.8	51.5	52.5
64	Agile gibbon	*Hylobates agilis*	2	Hyl	D	A	5.9	4.1	165.0	12.2	4.4
65	White-handed gibbon	*Hylobates lar*	2	Hyl	D	A	5.7	8.5	244.2	17.6	5.0
66	Mueller's gibbon	*Hylobates muelleri*	3	Hyl	D	A	2.9	4.1	188.6	47.6	3.5
67	Pileated gibbon	*Hylobates pileatus*	2	Hyl	D	A	6.7	6.8	198.0	7.2	4.0
68	Siamang	*Symphalangus syndactylus*	2	Hyl	D	A	11.0	9.0	183.2	45.3	3.5
69	Sumatran orangutan	*Pongo abelii*	3	Hom	D	A	60.0	6.8	275.6	49.2	2.0
70	Chimpanzee	*Pan troglodytes*	3	Hom	D	sA	41.3	19.4	272.0	54.3	53.0
71	Western gorilla	*Gorilla gorilla gorilla*	4	Hom	D	T	130.1	29.4	335.0	60.8	12.0
						Average	8.7	10.9	214.0	28.3	23.3
						SD	17.1	6.8	52.8	19.3	20.8
						CV%	196.9	62.3	24.7	68.2	89.1

Species names and scientific names are based on Rowe [42] and Groves [43].

1) Lem: Lemuridae; Lor: Loroidea; Cal: Callitrichidae; Ceb: Cebidae; Cer: Cercopithecidae; Hyl: Hylobatidae; Hom: Hominidae. Note that Loroidea is “superfamily-based” [42].

2) D: diurnal; N: nocturnal.

3) A: arboreal; sA: semiarboreal; T: terrestrial.

4) Group size data are based on Rowe [42], Smuts et al. [49], Campbell et al. [50], and Rowe and Myers [51].

### Ethical Statements

The present study adhered to the 2002 Version of the *Guide for the Care and Use of Laboratory Primates* by the Primate Research Institute, Kyoto University, and was approved by the Animal Welfare and Animal Care Committee of the Primate Research Institute, Kyoto University, and by the Animal Research Committee of Kyoto University. All the zoos studied are members of the Japanese Association of Zoos and Aquariums (JAZA), and daily care also adhered to the Ethical Guidelines of JAZA. Our study was conducted according to these guidelines and the *Ethical Guidelines for the Conduct of Research on Animals by Zoos and Aquariums* of the World Association of Zoos and Aquariums [44].

### Blink Recording

Video recorders (e.g., Handycam DCR-VX2000 and TRV-10; Sony, Tokyo, Japan) were used to record eye-blinks. Video recording was done between 09:30 and 17:00 in winter or until 18:00 in summer. Video recording was mainly done in daylight, although infrared lamps were used under dim light conditions when the individuals had returned to their sleeping quarters after dinner or when we recorded nocturnal primates. The accumulated recording time for each individual was at least 5 min because some studies have recommended that the minimum sampling time should be 3 min with the ideal time being 5 min for humans [39], [45]. Indeed, a previous study investigating the correlation between the blink rate and interblink interval indicated that a 5-min recording time is essential for humans [45]. Therefore, although the blink rate of other primates is generally lower than that in humans, we decided that 5 min of recording was appropriate.

### Data Reduction and Analysis

The videotaped eye-blink data were transferred to a PC using Adobe Premiere (Adobe Systems Co., Tokyo, Japan) and DVgate Plus (Sony) software. Data were reduced to obtain blink wave attributes, including amplitude, closing duration, reopening duration, and blink rate using a Blink Detection Program (Mizuno Measurement Co., Sendai, Japan), which was developed specifically for the present study. Using this program, we could precisely determine the necessary blink measures through playback of the video recording. Videos were viewed at slow speed (½ or ¼ of the original speed) because blink velocities of primates are generally high and it was possible to miss blinks at the normal playback speed and the same blink events may have been counted several times. Using the video recordings, we determined the following blink parameters: the number of blinks, the number of frames from the start to completion of each blink, and whether the blink was associated with specific body movements, including head and eye movements. Therefore, we obtained three different measures of blinks: the blink rate (bpm), blink duration (ms), and “isolated” blink ratio (%; proportion of blinks not associated with any type of body movement).

### Factors Contributing to Eye-Blinks

Three factors potentially linked to eye-blink behaviors [i.e., body size (body weight), activity rhythm, and habitat type] were selected based on previous reports [42], [43], [46]–[50]. Mean body weight values were determined for each species and averaged across males and females. Activity rhythm was classified as nocturnal (*n*  =  5) or diurnal (*n*  =  66). Habitat type was classified as arboreal (*n*  =  45), semiarboreal (*n*  =  19), or terrestrial (*n*  =  7). We also used the group size as a social factor. The mean group size for each species was calculated on the basis of Rowe [39], Smuts et al. [49], Campbell et al. [50], and Rowe and Myers [51].

## Results

### Overall Characteristics of Eye-Blink Behaviors in Primates

**Figure 1:**
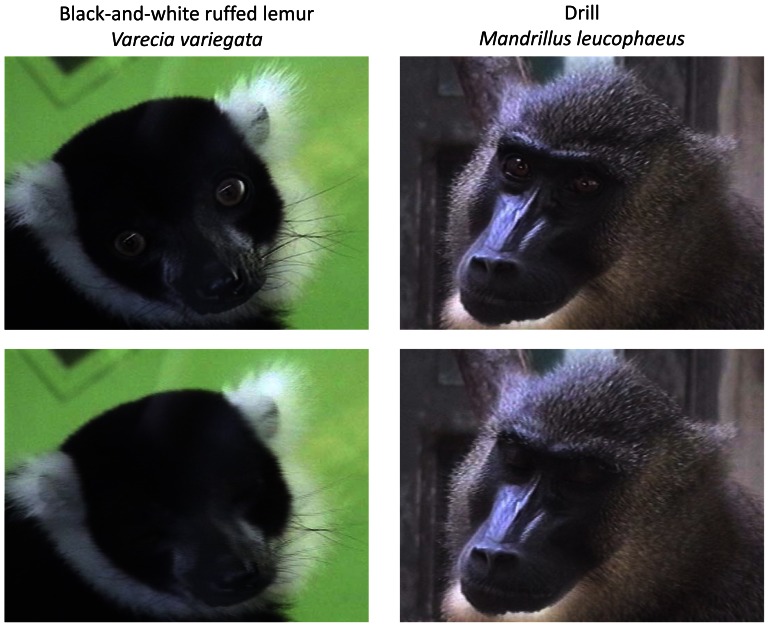
Two examples of eye-blinking behaviors in nonhuman primates. Left: Black-and-white ruffed lemur (*Varecia variegata*); right: the drill (*Mandrillus leucophaeus*).

Figure 1 shows two examples of eye-blinks by the primate species the black-and-white ruffed lemur (*Varecia variegata*) and drill (*Mandrillus leucophaeus*). Data for each species are shown in Table 1. The mean values for eye-blink measures and body size and group size measures for 141 individuals of 71 species are given at the bottom of Table 1. The mean ± SD blink rate was 10.9 ± 6.8 bpm and was highest in white-fronted capuchin (*Cebus albifrons*; 29.8 bpm) and western gorilla (*Gorilla gorilla*; 29.4 bpm), and lowest in potto (*Perodicticus potto*; 0 bpm), lesser slow loris (*Nycticebus pygmaeus*; 0.2 bpm), and brown greater galago (*Otolemur crassicaudatus*; 0.3 bpm). The potto did not blink during the 5-min recordings. The mean blink rate was approximately half of that in humans, which was 20.8 bpm in our own earlier study performed in approximately 1400 adults [24] and as reported elsewhere [6], [7], [52]. However, the variation in blink rate was fairly large, with SD and coefficient of variation (CV) values of 6.8 bpm and 62.3%, respectively. In our earlier study, the respective SD and CV values in humans were 14.7 bpm and 70.7% [24].

The mean blink duration of the 71 species of primates was 214.0 ± 52.8 ms (CV  =  24.7%). Blink duration was longest in the brown greater galago (341.0 ms), western gorilla (335.0 ms), and black-and-white ruffed lemur (323.4 ms), and shortest in the Guinea baboon (*Papio papio*; 138.6 ms), Goeldi's marmoset (*Callimico goeldii*; 141.9 ms), and Bolivian squirrel monkey (*Saimiri boliviensis boliviensis*; 148.5 ms). In comparison, the blink duration and CV in humans were 403.6 ± 52.6 ms and 13.0% [21], respectively. Thus, the blink duration and CV in primates were approximately half and about double those in humans, respectively.

The mean isolated blink ratio in primates was 28.3% ± 19.3%, and was highest in the lesser slow loris (100%), Sumatran orangutan (*Pongo abelii*; 70.6%), and Tibetan macaque (*Macaca thibetana*; 68.9%), and lowest in the grand galago (*Galago crassicaudatus*; 0%), black-capped squirrel monkey (*Saimiri boliviensis*; 0%), and ring-tailed lemur (*Lemur catta*; 0%). We do not yet have sufficient data to determine the isolated blink ratio in humans, but from our experience, the isolated blink ratio in humans, at least under experimental conditions, is high in adults. Based on limited data obtained from 408 subjects, including very young babies [24], the isolated blink ratio seems to increase with age, particularly in young children.

### Effect of Activity Rhythms

For subsequent analyses, we transformed all of the data into common logarithmic values for data analyses, although retransformed values are presented in the text.

**Figure 2:**
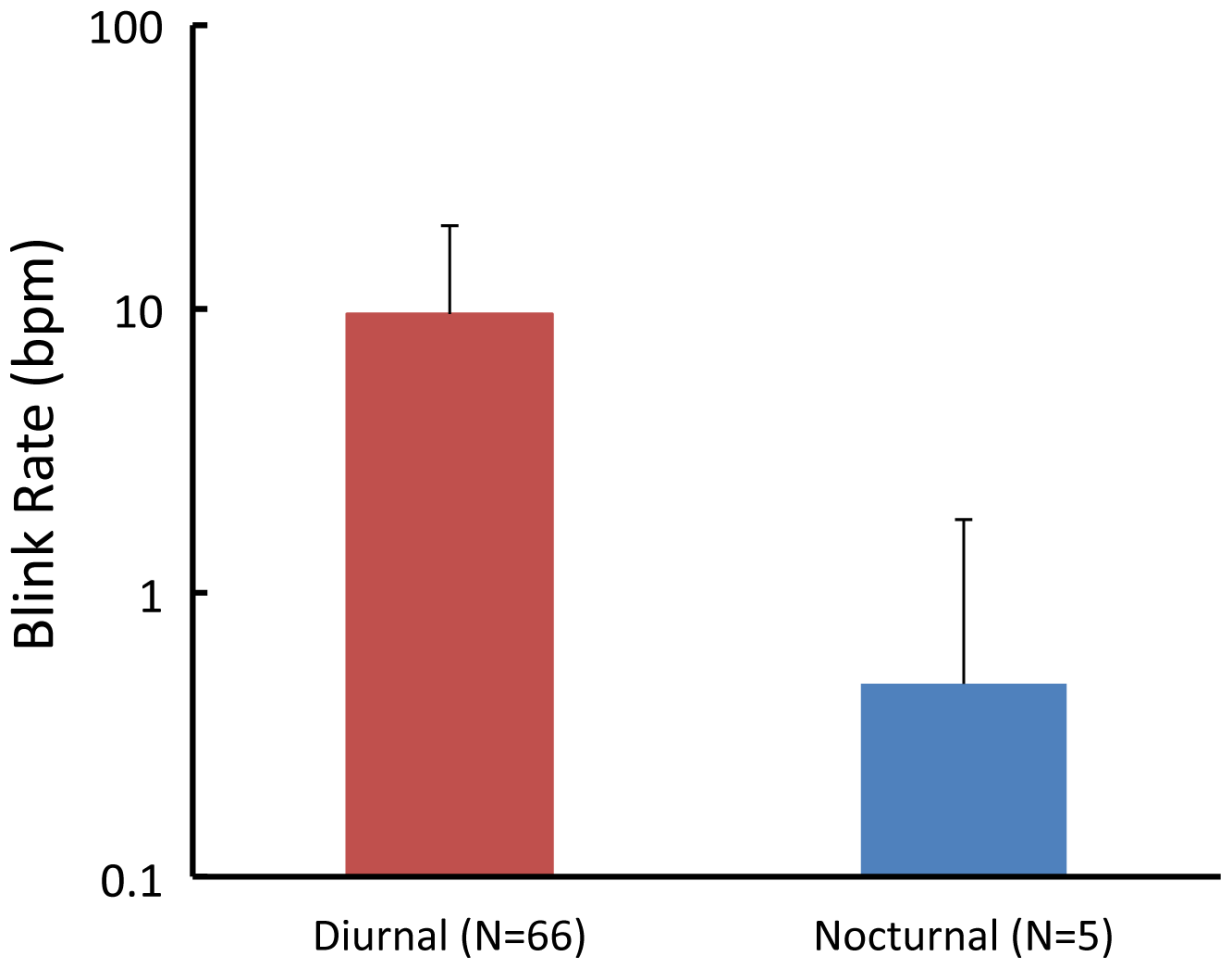
Mean blink rate as a function of activity rhythms. Error bars show standard deviations.

First, we examined the effects of activity rhythms on eye-blink behaviors, although analyses of blink duration and isolated blink ratio were not conducted because of the small numbers of species. Figure 2 shows the mean blink rate as a function of activity rhythms. Diurnal primates exhibited more eye-blinks than nocturnal primates [9.62 vs. 0.47; *t*(68)  =  7.74, *P* < 0.001]. The results of multiple regression analysis using activity rhythms as dummy variables to control for body size factors indicated that this difference was still significant [*R* ^2^  =  0.515, *F*(2,67)  =  35.59, *P* < 0.001; regression coefficient for body weights  =  0.169, *P*  =  0.013; regression coefficient for activity rhythm  =  1.167, *P* < 0.001]. Based on these results, subsequent analyses focused only on the data from diurnal primates.

### Effects of Habitat Type

**Figure 3:**
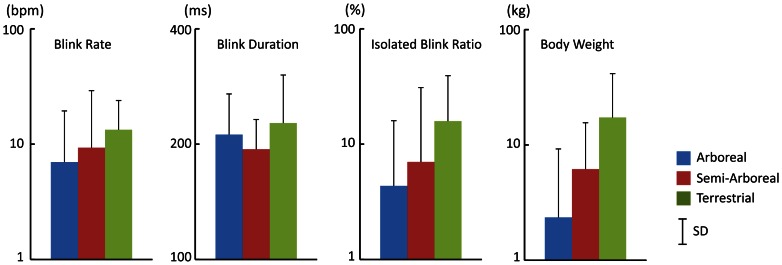
Three eye-blink measures and body weight as functions of habitat type. SD: standard deviation.

Figure 3 shows the mean blink rate, blink duration, isolated blink ratio, and body weight as a function of habitat type. Some correlations were observed between these three measures and habitat types. However, body size factors were also correlated with habitat type. We also conducted multiple regression analyses for each measure using body size and habitat type as independent variables. Three habitat types were used as dummy variables. Table 2 shows the results of multiple regression analyses. For the blink rate, no significant multiple regression were noted, whereas regressions were significant for the other two measures. In these two measures, however, only the regression coefficients for body weight were significant. These results clearly indicated that habitat types do not affect eye-blink behaviors.

**Table 2:** Results of multiple regression analyses on body size and habitat type factors.

					Regression Coefficients			
							Habitat (Reference = Arboreal)	
Measures	R2			Intercept	Body Weight		Semi-Arboreal	Terrestrial
Blink Rate	0.115	*F*(3,62) = 2.69		2.483	0.128		0.097	0.102
Blink Duration	0.142	*F*(3,60) = 3.32*		2.100	0.063	*	−0.054	−0.010
Isolated Blink Ratio	0.234	*F*(3,60) = 4.12***		0.596	0.210	**	−0.015	0.114

* : *P*<0.05, **: *P*<0.01, ***: *P*<0.001.

### Effects of Group Size

**Figure 4:**
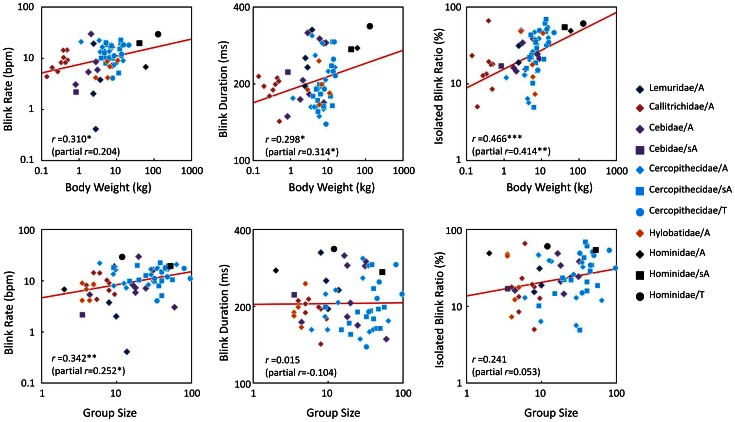
Scatterplots of the three measures for eye-blinking behaviors in primates as a function of body size and group size factors. Red lines indicate the simple regression lines. ***: *P* < 0.001, **: *P* < 0.01, *: *P* < 0.05. A: arboreal; sA: semiarboreal; T: terrestrial.

**Figure 5:**
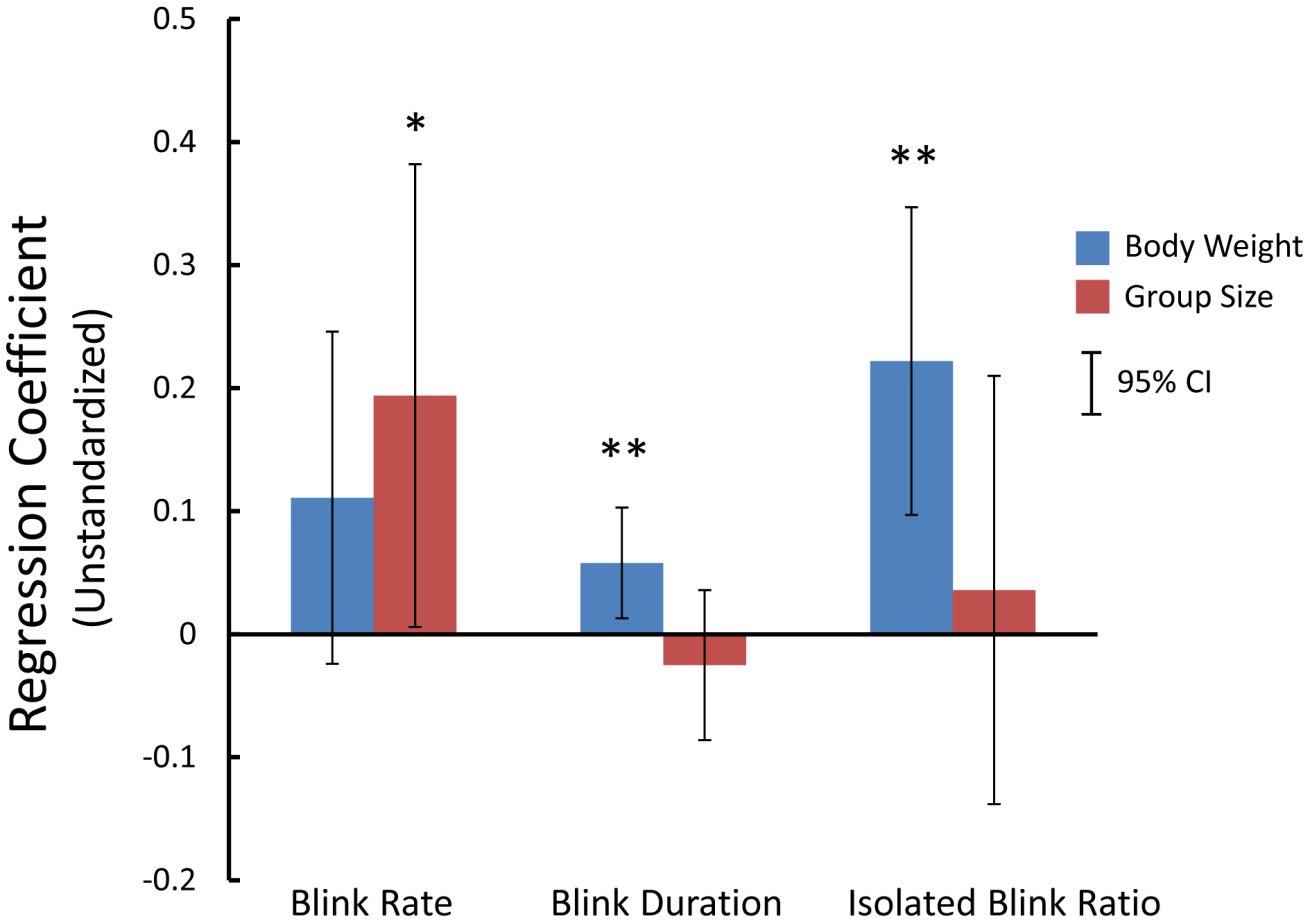
Regression coefficients for body weight and group size factors for each eye-blink measure. Error bars show 95% confidence intervals.

Figure 4 shows scatterplots of three measures against body weight and group size. Simple correlation coefficient and simple regression line are shown in each panel. These three eye-blink measures were significantly correlated with body weight. Group size, however, was only significantly correlated with blink rate, as shown in Figure 4. For further investigation, multiple regression analyses with body weight and group size as independent variables were conducted for these data. The results indicated significant multiple regressions for blink rate [*R* ^2^  =  0.154, *F*(2,63)  =  5.73, *P*  =  0.005], blink duration [*R* ^2^  =  0.099, *F*(2,61)  =  3.34, *P*  =  0.042], and isolated blink ratio [*R* ^2^  =  0.181, *F*(2,62)  =  6.83, *P*  =  0.002]. Note that the contributions of each independent variable varied across eye-blink measures. Figure 5 shows the unstandardized regression coefficients for each variable. As indicated in this figure, the effect of body weight was significant for blink duration and isolated blink ratio, while the group size significantly affected the blink rate only. Figure 4 also shows partial correlation coefficients in parentheses after partialling out the other variable.

## Discussion

The results of this study can be summarized as follows. First, eye-blink behaviors in primates are affected by the activity rhythms (especially with regard to blink rate), but not habitat type. The effects of activity rhythms on spontaneous blinks were reported previously in nonhuman animals, including other primates [13]. Our results clearly replicated the previous results. Furthermore, these tendencies are not “virtual,” as the effect remained after partialling out body size factors.

Second, group size factors also affected eye-blink behaviors, but the effects varied across behavioral measures. Blink duration and isolated blink ratio are correlated with body weight but not with group size, an important social factor [1]. As in the other allometric parameters, such as locomotor speed and life span [53], [54], variations in these two measures were simply explained by the body size factors. In contrast, however, the blink rate was not correlated with body weight but with the group size. This result is quite suggestive. From a physiological perspective, a rate of 2 bpm has been reported to provide sufficient corneal wetting for human adults [55], [56]. Regardless of whether this basic rate is constant against body size factors, the observed blink rate was clearly higher than this rate in 65 of 66 diurnal primates. Our data suggest that these “additional” blinks may be related to social factors. Dunbar [1] reported that group size is significantly correlated with the neocortex ratio (i.e., the ratio between the volume of the neocortex and the rest of the brain) after partialling out body weight factors. Their results are considered as evidence for the “social brain hypothesis” or “social (Machiavellian) intelligence hypothesis” [1]–[3]. The observation that neocortex size, a simple indicator of the cognitive capabilities, was correlated with group size, a simple indicator of complexity of social lives, suggests that a large brain (neocortex) evolved as a result of social complexity.

Many reports have been published regarding the relationship between the effect of group size and related factors, and social and visual behaviors in primates. For example, Dunbar reported that the group size was significantly positively correlated with time spent in social grooming in nonhuman primates [57]. With regard to vigilance or visual monitoring behavior, Kutsukake reported that vigilance duration in chimpanzees increased significantly as a function of the number of individuals [58]. Vigilance is not simply governed by external threats, such as predators, but by conspecific factors in chimpanzees. A similar tendency was observed in capuchins [59], but the reversed tendency was also reported in nonprimate species, such as ungulates [60]. Furthermore, Kobayashi and Hashiya [61] reported that wide eye-shape in primates, including humans, was also correlated with group size. Humans have the widest eyes among primates and are the only species with white sclera [38]. Kobayashi and colleagues also found that the rate of gaze behavior without head or body motion was correlated with the width of the eyes [38] and group size [61], and suggested that gaze may play the role in “remote” grooming.

Taken together, these previous findings suggest that eye-blinks, a looking behavior along with vigilance and gazing, may be governed by social factors. For monitoring to detect inter- or intraspecific risks, vigilance behavior should increase with increases in potential threats, as shown previously [58]–[60]. Blinking, however, shuts down external information even though it is for a very short duration. Humans are known to show greater difficulty in detecting changes in scenes during blinking (i.e., change blindness [62], [63]), and spontaneous eye-blinks are highly synchronized with scene breaks when watching videos to minimize the chance of losing critical information [64]. If primates increased monitoring activity, the blink rate would have decreased to maintain a higher vigilance level as the group size increased. Human pilots in flight simulators were shown to blink more frequently and with longer durations when flying over “friendly” (less vigilant) skies than when flying over “enemy” territory [65]. Furthermore, when they were targeted by enemy radar, the pilots blinked very infrequently and the blinks were of very short duration [65]. Under some specific vigilant contexts, eye-blink rates actually decreased in humans. Our results, however, showed the opposite tendency, i.e., the blink rate increased as a function of group size. Thus, an increased blink rate may have a different or additional social role to visual monitoring. One candidate role may be for social communication, similar to grooming or gazing [57], [61]. Eye-blink behavior may have developed as a means of remote visual communication. The observation that the blink rate affects the formation of a person's impression in humans is a suggestive example of eye-blinking as a social communication tool [66]. This hypothesis may also be indirectly supported by the “colorful” primates [67]. Some species of primates (e.g., mangabeys, baboons, and some species of macaques) have very colorful faces and especially bright white eyelids [67]. Some studies have suggested that this coloration may have evolved to adapt to visually “noisy” environments [68]. However, bright color around the eyes may enhance attention to the eye region, as in the human sclera. Furthermore, this eyelid color is most enhanced when the monkeys are blinking, and this coloration may be indicative of the use of eye-blinks for social communication. Although whether species with such highlighted eyelids show more eye-blinks than other species is unclear, our preliminary data suggest that species with contrasting bright eyelids (mangabeys, gelada baboons) show lower blink rates (9.9 bpm) than other species (genus *Macaca*, family Cercopithecidae, 14.5 bpm, *n*  =  11); this difference, however, was not significant [*t*(12)  =  1.44, *P*  =  0.174].

In summary, we found significant relationships between some aspects of eye-blink behaviors (blink rate) and group size, as well as body size and activity rhythm factors. In addition, we propose that spontaneous eye-blinks in primates have some role in social communication. Further studies are required to test this possibility under more controlled settings. For example, our data on blink rate were correlated with the “average” group size. As in the case for vigilance behavior in the study by Kutsukake [58], one will have to examine how eye-blink behaviors in primates are affected by the “actual” social contexts of each individual, such as the number of spatially proximate conspecifics. Also, investigating the developmental origin of eye-blink behaviors from the standpoint of comparative cognitive science will be necessary [69]. Humans show increased blink rate from the minimum level to around 20 bpm during the course of development [6], [7], [24], [52]. Unfortunately, no data concerning the development of eye-blink behaviors in nonhuman primates are available. Spontaneous eye-blinking is affected by cognitive demands [64], [70], [71]. One will need to examine how the varied cognitive demands affect the eye-blink behaviors in various primates. We also emphasized the relationship between facial (especially eyelid) coloration of some primate species and eye-blink behavior, although definitive conclusions cannot yet be reached based on our results. Further detailed observations will provide more insight into the social role of eye-blinks in primates, including humans.
